# Case of Alleged Uterine Respiration

**Published:** 1846-11

**Authors:** 


*Case of alleged Uterine Respiration.*—In November, 1845, M. Tourtois was called to attend a female in labour, who had already borne several children. The membranes had become ruptured, and the waters had been discharged about an hour before his arrival. The head was descending into the pelvis, and in attempting to give it a better direction, M. Tourtois introduced two fingers into the child's mouth, when, to his surprise, he felt them sucked or drawn in with some force. Others who were present satisfied themselves of the existence of this phenomenon; and at intervals for more than half an hour, the child, which thus breathed before birth, continued to suck the finger of the accoucheur with great energy. The woman, after some hours, was delivered of a lively female child, which sucked the breast vigorously an hour after its birth.—*Gaz. Med.*

[Admitting that the act of sucking was real, and that air was drawn into the lungs of the child, it will be observed that the rupture of the membranes, the decent of the head, and the introduction of the hand of the accoucheur, all tended to favour the imperfect act of respiration before birth. To apply to it the term uterine respiration, is, under these circumstances, quite inappropriate.]—*Lond. Med. Gaz.*

